# PARP inhibition preserves cone photoreceptors in *rd2* retina

**DOI:** 10.1186/s40478-025-01982-5

**Published:** 2025-04-01

**Authors:** Pakize Nur Akkaya, María Miranda, Inmaculada Almansa, Cigdem Elmas, Dragana Trifunovic, Zohreh Hosseinzadeh, Ayse Sahaboglu

**Affiliations:** 1 Department of Histology-Embryology, Balikesir University Faculty of Medicine, Balikesir, Türkiye; 2 Department of Ophthalmology, Radboud University Medical Center, Nijmegen, Netherlands; 3 Departamento Ciencias Biomédicas, Facultad de Ciencias de La Salud, Universidad Cardenal Herrera-CEU, CEU Universities, Valencia, Spain; 4 Department of Histology-Embryology, Gazi University Faculty of Medicine, Ankara, Türkiye; 5 Centre for Ophthalmology, Institute for Ophthalmic Research, Eberhard Karls University, Tübingen, Germany

**Keywords:** *rd2* retinas, Photoreceptor cell death, PARP inhibition, Cone degeneration, Retinal inflammation

## Abstract

The *rd2* mouse model, characterized by a mutation in the *Prph2* gene, exhibits abnormal development of photoreceptor outer segments, resulting in progressive retinal degeneration. While the correlation between poly-ADP-ribose polymerase (PARP) activity and the degeneration of rod photoreceptors is established in the *rd2* model, the specific mechanism driving cone degeneration in this model remains unclear. Furthermore, it is yet to be determined whether inhibiting PARP activity can effectively impede the degeneration of cone photoreceptors in this context. We demonstrated that PARP inhibitors Olaparib, BMN-673, and 3-aminobenzamide (3AB), effectively reduced photoreceptor cell loss in the *rd2* retina. Notably, *rd2* retinas exhibited decreased cone density, but treatment with PARP inhibitors significantly protected cone photoreceptors. The PARP inhibitors, particularly BMN-673, demonstrated a significant protective effect as evidenced by increased rhodopsin expression within the outer segment and a concurrent decrease in Müller cell activity indicated by GFAP expression. The treatment also resulted in significant changes for markers of oxidative stress, such as glutathione (GSH), and oxidized glutathione (GSSG). Notably, the administration of PARP inhibitors also reduced CD9 expression (extracellular vesicle marker), which were significantly increased within the outer nuclear layer (ONL) in the *rd2* retinas. Among PARP inhibitors, BMN-673 demonstrated the highest efficacy in preserving photoreceptors, particularly benefiting cone cells.

**Graphical Abstract:**

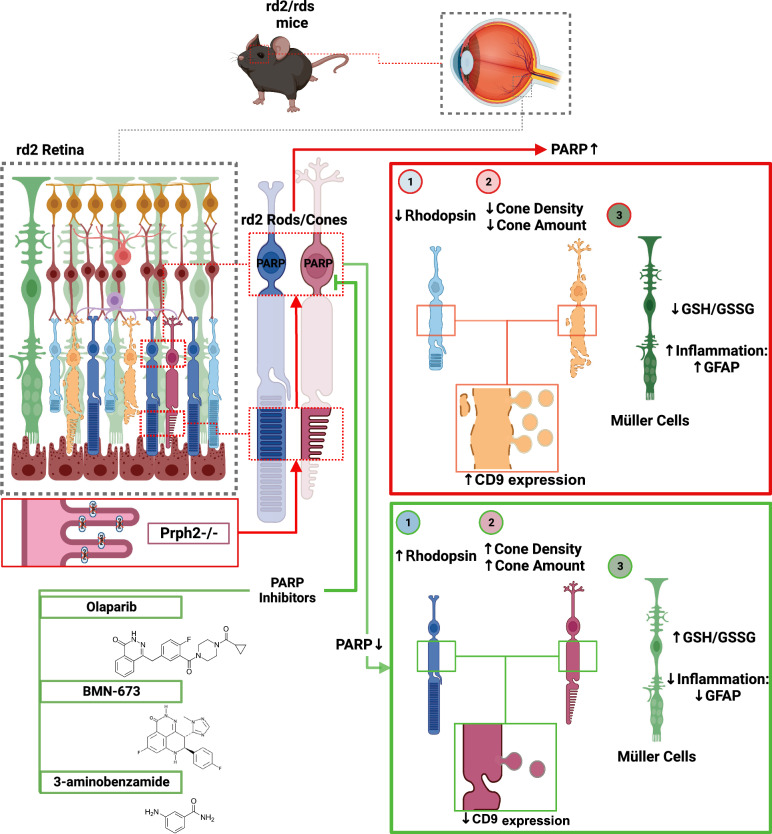

**Supplementary Information:**

The online version contains supplementary material available at 10.1186/s40478-025-01982-5.

## Introduction

During retinal development, inherited retinal degenerations (IRDs) can develop due to various genetic mutations involving more than 300 genes [1] (https://sph.uth.edu/RetNet; information retrieved November 2023). Mutations in the *PRPH2* gene in human cause IRDs by disrupting the outer segment structure, leading to slow and progressive rod and cone photoreceptor degeneration [1, 2]. In rodents, the development and differentiation of photoreceptors typically concludes between 10 and 30 postnatal days [3]. However, in the *rd2* mouse model, bearing a mutation in the *peripherin/rds* gene (*Prph2*), exhibits a lack of outer segment formation and undergoes slow yet progressive photoreceptor degeneration [4]. In the *rd2* mouse model, the mutation leads to failure in the development of photoreceptor outer segments (OSs), leading to the incomplete electroretinographic response, which gradually diminishes over time [5]. This phenotype observed in the *rd2* mouse closely parallels the situation found in human patients, establishing the *rd2* mouse as a suitable and pertinent model for conducting pathophysiological and therapeutic studies [5]. A mutation in the* PRPH2* gene commonly causes severe retinopathies such as retinitis pigmentosa (RP), Leber’s congenital amaurosis, or cone dystrophies [6, 7]. Cone degeneration can be divided into three groups: primary mutation-dependent cone degeneration [8], secondary cone degeneration following mutation-dependent primary rod degeneration [9], and mutation-dependent and independent rod-cone degeneration under stress conditions [10–12]. All these cone degenerations are associated with different diseases: primary cone degeneration with achromatopsia, secondary cone degeneration with RP, and rod-cone degeneration with age-related macular degeneration (AMD) [13, 14]. RP is a group of genetic disorders that affect the photoreceptor cells, leading to progressive vision loss. While mutations in the *PRPH2* gene, which codes for the peripherin-2 protein, are associated with some cases of autosomal dominant RP, it’s essential to note that RP is a genetically heterogeneous condition. RP triggers mutation-dependent rod photoreceptor cell death, followed by cone photoreceptor cell death driven by a pathway that is not yet fully understood [15]. Such secondary cone degeneration can be surprisingly slow, referred to as “slow retinal degeneration” [10]. Incomplete outer segment development leads to pathophysiological processes affecting rod and cone photoreceptors. As cone photoreceptors require more energy than rod photoreceptors [16], their high demand for ATP may contribute to the progression of cone cell death. Another retinal disease associated with cone alterations is AMD. Cone photoreceptors are disorganized at the fovea or parafovea in patients with early AMD [17]. Several functional assessments, including focal electroretinogram (ERG), multifocal ERG, and cone adaptation, suggest that cone function is impaired in the early stages of the disease [18]. Although it is still not fully understood how cone photoreceptor loss develops in RP and macular degenerations such as AMD, increased PARP activation appears to be a common factor in both diseases [19, 20].

PARP is an enzyme that catalyzes oligo- and poly-ADP-ribosylation of proteins such as histones, DNA polymerases, topoisomerases, and transcription factors or directly from NAD + [21–23]. It is involved in critical cellular events such as DNA repair, maintenance of genomic stability, transcriptional regulation, energy metabolism, histone acetylation and methylation, and cell death [24, 25]. At least 17 different PARP isoforms are known and PARP1 isoform plays a fundamental role in cell physiology [19, 23]. Mild DNA damage activates PARP1, leading to repair damage, but severe DNA damage leads to overactivation of the PARP1 enzyme, initiating cell death [20, 24]. Excessive PARP activity leads to increased consumption of NAD + , intense consumption of ATP, energetic collapse, and cellular dysfunction. This results in the accumulation of polyADP ribose (PAR) and the translocation of apoptosis-inducing factor (AIF) from mitochondria to the nucleus [24, 25]. The PARP-dependent but caspase independent mechanism of cell death has been termed ‘PARthanatos’ [24]. The importance of PARP in cellular physiology has led to the use of PARP inhibitors in the treatment of certain cancers, including ovarian, breast, and prostate malignancies [26–29], as well as pre-clinical and clinical trials for neurodegenerative diseases [30–32], including retinal diseases [30–32]. In this study, we explore the repurposing potential of approved PARP inhibitors: Olaparib, and BMN-673 as well as 3AB PARP inhibitor currently undergoing preclinical evaluation to assess its impact on cone protection. PARP activity-dependent oxidative stress in retinal injury has been investigated [33, 34]. PARP enzyme and PAR accumulation increase in parallel with the peak of photoreceptor death in retinal degeneration models, including the *rd2* model [19]. Previous studies have shown that, PARP inhibition protects against primary rod degeneration [35–37]. In addition, a novel PARP1 inhibitor has been demonstrated as a therapeutic option for dry AMD [20, 38]. However, the effect of PARP inhibition particularly on cone photoreceptors and its potential to prevent cones degeneration, has remained unknown.

In this study, we presented a new insight into cone degeneration in the *rd2* model. Cone photoreceptor density was decreased in the *rd2* mouse model. We also compared the rate of cone and rod degenerations in *rd2* retinas. We demonstrated the efficacy of PARP inhibition in protecting cone photoreceptors. Finally, we provided a novel mechanism of PARP inhibition-dependent neuroprotection resulting in reduced CD9 expression, Müller cell activation and oxidative stress markers. The reduction of cone photoreceptor degeneration by PARP inhibition provides a new perspective for elucidating the therapeutic approaches in retinal degeneration.

## Results

### PARP inhibition reduces PARylation in *rd2* photoreceptors

**Fig. 1:**
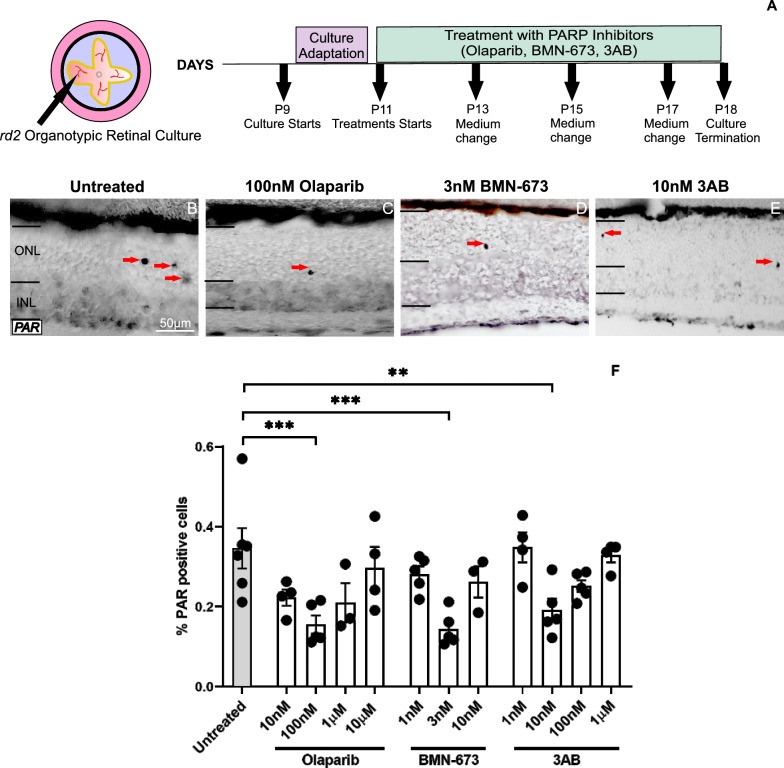
Treatment schedule of *rd2* organotypic retinal culture and PARP inhibition decreases PARylation. A schematic figure of *rd2* organotypic retinal culture treatments with Olaparib, BMN-673, and 3AB (**A**), PAR immunoreactivity in untreated *rd2* retinas (**B**), as well as Olaparib (**C**), BMN-673 (**D**), and 3AB (**E**) treated groups. Scale bar, 50 μm. The plots show the percentages of PAR positive photoreceptors in the outer nuclear layer (ONL) (**F**). Arrows indicate PARylated photoreceptors. The images shown represent observations on at least three different specimens for each genotype/treatment condition. *n* ≥ 3, significance levels: *P < 0.05, **P < 0.01, ***P < 0.001. ANOVA testand Kruskal–Wallis test for multi-comparisons

Our previous research has shown that overactivation of PARP leads to PAR accumulation within the *rd2* retina [19]. In this study, DAB staining was used to identify PARylation in photoreceptors. The effects of different PARP inhibitors, namely Olaparib, BMN-673, and 3AB at different concentrations, were used to treat in vitro * rd2* organotypic retinal cultures for one week (Fig. 1A). Olaparib was administered at concentrations of 10 nM, 100 nM, 1 µM, and 10 µM; BMN-673 at concentrations of 1 nM, 3 nM, and 10 nM; and 3AB at concentrations of 1 nM, 10 nM, 100 nM, and 1 µM respectively (Fig. 1B–F). Notably, the groups treated with 100 nM Olaparib, 3 nM BMN-673, and 10 nM 3AB showed a statistically significant decrease in PARylation levels compared to untreated retinas*.* (untreated: 0.34 ± 0.05 SEM *n* = 6, 100 nM Olaparib: 0.15 ± 0.02 SEM *n* = 5 p = 0.0007, 3 nM BMN-673: 0.14 ± 0.01 SEM *n* = 5 *p* = 0.0003, 10 nM 3AB: 0.19 ± 0.02 SEM n = 5 *p* = 0.0076) (Fig. 1B–F). These findings underscore PAR-related dysregulation in pathologies of the *rd2* retina.

### PARP inhibition decreases photoreceptor cell death in *rd2* retinas

In our study, we selected two approved inhibitors, Olaparib and BMN-673, to facilitate translation into clinical trials for peripherin-2-related retinal diseases, as well as 3-aminobenzamide, previously shown to be a potent PARP inhibitor. The concentrations were chosen based on our previous experience with different degeneration models and IC50 values. It is crucial to note that within the nanomolar range, 3-aminobenzamide may inhibit mono ADP-ribosylation [39], suggesting a potentially more effective role in the inhibition of PARP activity.

**Fig. 2:**
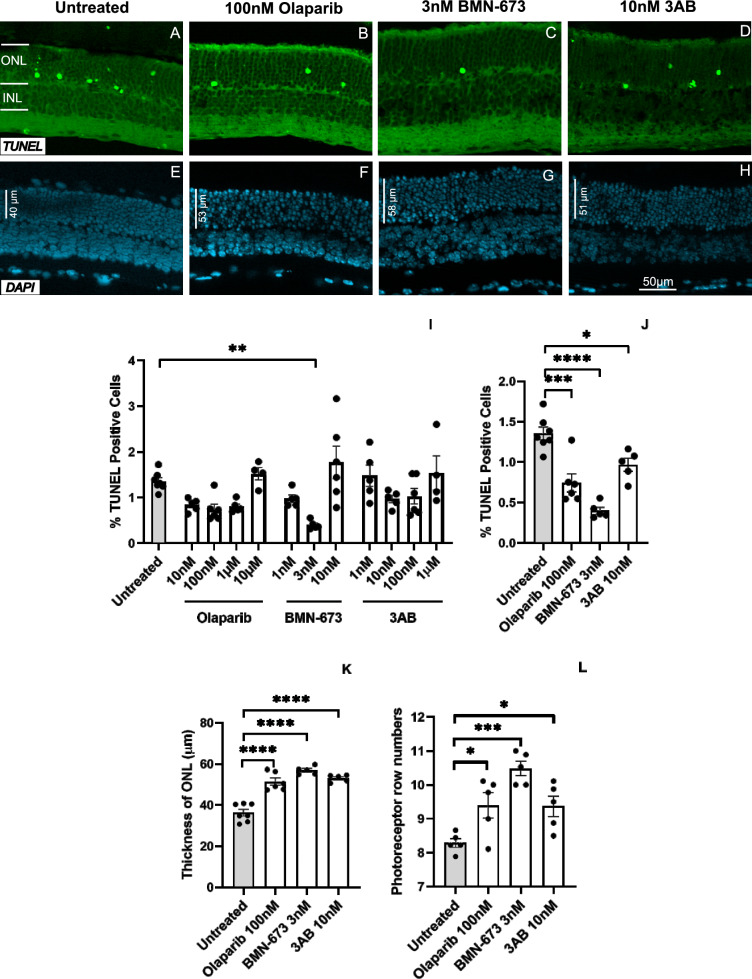
PARP inhibition prevents photoreceptor cell death in the *rd2* retina. TUNEL assay showed a significant decrease in the numbers of positive cells for 100 nM Olaparib, 3 nM BMN-673, and 10 nM 3AB treated groups in the organotypic retinal culture. The most neuroprotective PARP inhibitor was 3 nM BMN-673 (**A–D, I, J**). The thickness and photoreceptor row number were increased for all three inhibitors (**E–H, K, L**). The images shown are representative for observations on at least three different specimens for each genotype/treatment condition. *n* ≥ 3, significance levels: *p < 0.05, **p < 0.01, ***p < 0.001, ****p< 0.0001. ANOVA test and Dunnett’s test for multiple comparisons

Our current study investigated the effect of different concentrations of Olaparib, BMN-673, and 3AB on *rd2* retinal degeneration. The results highlight those specific concentrations of these PARP inhibitors showed remarkable effects in reducing the dying cell population. Concentrations of 100 nM for Olaparib, 3 nM for BMN-673, and 10 nM for 3AB were found to be the most effective in reducing cell death within the *rd2* organotypic retinal cultures (untreated: 1.35 ± 0.07 SEM *n* = 7, 100 nM Olaparib: 0.74 ± 0.11 SEM *n* = 6 *p* = 0.0001, 3 nM BMN-673: 0.4 ± 0.04 SEM *n* = 5 *p* < 0.0001, 10 nM 3AB: 0.96 ± 0.08 SEM *n* = 5 *p* = 0.012) (Fig. 2A–D, J). According to these results, 3 nM BMN-673 (untreated: 1.35 ± 0.07 SEM *n* = 7, 3 nM BMN: 0.4 ± 0.04 SEM *n* = 5 *p* < 0.0001) was the most effective PARP inhibitor with the maximum effect in reducing photoreceptor cell death (Fig. 2I).

Measurement of photoreceptor layer thickness and row numbers reflects the efficacy of potential treatments for retinal diseases. Therefore, we analyzed ONL thickness and photoreceptor row numbers in different treatment conditions. ONL thickness and photoreceptor row numbers were significantly increased in groups treated with 100 nM Olaparib, 3 nM BMN-673, and 10 nM 3AB. These results suggest that these specific concentrations of Olaparib, BMN-673, and 3AB have a positive effect on the structural integrity of the ONL in *rd2* organotypic retinal cultures. This increase in ONL may have the potential to preserve photoreceptors, highlighting the therapeutic potential of these PARP inhibitors in retinal degeneration (ONL thickness, untreated: 36.29 ± 1.62 SEM *n* = 7, 100 nM Olaparib: 51.40 ± 1.8 SEM *n* = 6, 3 nM BMN-673: 56.87 ± 8.85 SEM *n* = 5, 10 nM 3AB: 53.08 ± 7.55 SEM *n* = 5 *p* < 0.0001; photoreceptor row numbers, untreated: 8.29 ± 0.12 SEM *n* = 5, 100 nM Olaparib: 9.40 ± 0.37 SEM *n* = 5, 3 nM BMN-673: 10.48 ± 0.21 SEM *n* = 5, 10 nM 3AB: 9.37 ± 0.30 SEM n = 5 *p < 0.05, ***p < 0.001) (Fig. 2E–H, K, L).

### Cone photoreceptor density is decreased in *rd2* retinas

**Fig. 3:**
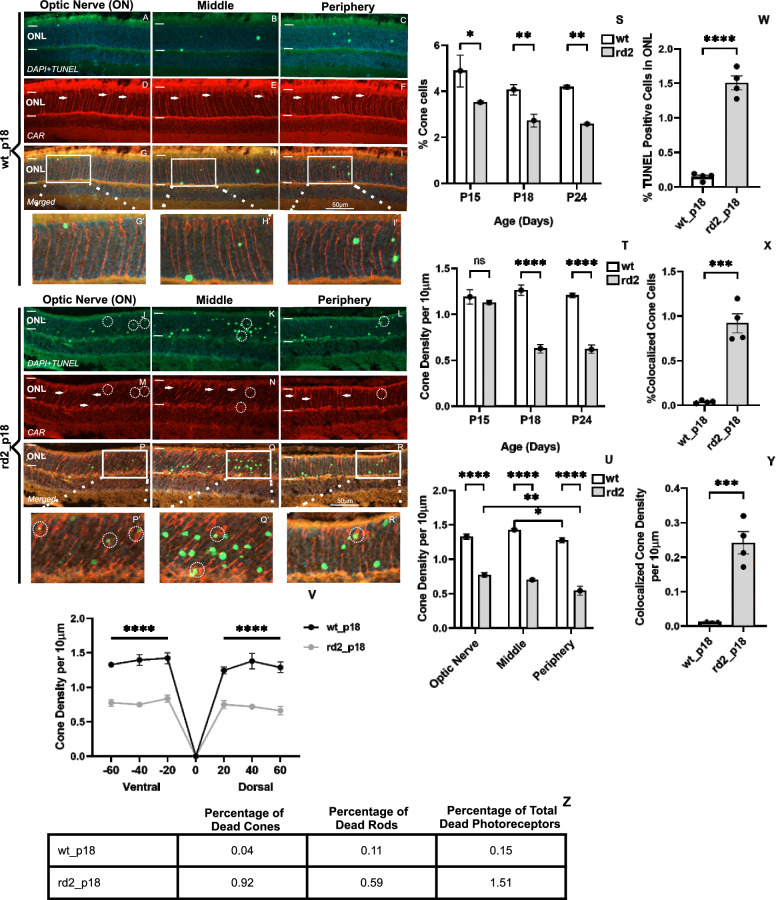
Density and percentage of cone photoreceptors in *rd2* retinas. DAPI, TUNEL and Cone-Arrestin (CAR) staining DAPI, TUNEL, and Cone-Arrestin (CAR) stainings in *wt* retina (**A–I’**) and *rd2* retinas (**J-R’**). Arrows indicate cones, and circles indicate colocalized cones stained with TUNEL, CAR, and in the merged image. Both the percentage and density of cone photoreceptors were decreased in ONL of *rd2* retinas compared to corresponding *wt* retinas at P15, P18, and P24 (**S, T**). The density of cone photoreceptors per 10 µm area was decreased in both the ventral and dorsal area (**V**) as well as in the peripheral, middle, and central to the optic nerve in *rd2* retinas (**U**). The total percentage of TUNEL-positive photoreceptors in the *rd2* retinas at P18 was significantly increased (**W**). The percentage and density of dead cone photoreceptors in the *rd2* P18 retinas were higher than the percentage of dead rod photoreceptors (**X, Y**). The table represents the percentage of dead cones, rods, and total photoreceptors (**Z**). Circles indicate representative cone photoreceptors. The images shown are representative for observations on at least three different specimens for each genotype. *n* ≥ 3, significance levels: *p < 0.05, **p < 0.01, ***p < 0.001, ****p< 0.0001. One-way and two-way ANOVA tests with Tukey’s test for multiple comparisons. Unpaired *t*-tests

This study focuses on understanding cone photoreceptors under physiological and pathophysiological conditions. The percentage of cone photoreceptors is often used in research and clinical studies to describe the relative composition of photoreceptors. However, cone photoreceptor density is useful for understanding the spatial arrangement of cones and their functional implications in vision. Therefore, we first measured the percentage and density of cone photoreceptors in the ONL in vivo preparations and in vitro organotypic retinal cultures. We determined the optic nerve (ON) as the zero point and analyzed the dorsal and ventral retina by dividing them into three parts: optic nerve (ON), middle, and periphery. Then, we examined the cone photoreceptor density in the wild-type(*wt)* and *rd2* retinas. As Fig. 3S indicates, the percentage of cone photoreceptors was significantly decreased in the *rd2* retinas at P15, P18 (peak of retinal degeneration), and P24 compared to corresponding *wt* retinas (*wt* p15: 4.88 ± 0.68 SEM n = 3, *rd2* p15: 3.51 ± 0.06 SEM *n* = 6 *p* = 0.0446, *wt* p18: 4.07 ± 0.22 SEM n = 6, *rd2* p18: 2.72 ± 0.28 SEM *n* = 9 *p* = 0.0011, wt p24: 4.18 ± 0.08 SEM *n* = 6, *rd2* p24: 2.57 ± 0.05 SEM *n* = 3 *p* = 0.0039). Similarly, analysis of cone density showed significant decrease in cone density in *rd2* retinas at P18 and P24, as Fig. 3T shows (wt p15: 1.19 ± 0.07 SEM n = 3, *rd2* p15: 1.13 ± 0.02 SEM *n* = 6 *p* = 0.9702, *wt* p18: 1.26 ± 0.05 SEM *n* = 6, *rd2* p18: 0.68 ± 0.04 SEM *n* = 9 *p* < 0.0001, *wt* p24: 1.20 ± 0.02 SEM n = 6, *rd2* p24: 0.61 ± 0.04 SEM *n* = 3 *p* < 0.0001). When we also examined the regional distribution of cone photoreceptor densities in *rd2* retinas, the density of cone photoreceptors in the *rd2* retina was mainly reduced in the periphery (ON: 0.77 ± 0.03 SEM *n* = 5 *p* = 0.5214, middle: 0.70 ± 0.02 SEM *n* = 5 *p* = 0.0091, periphery: 0.54 ± 0.06 SEM *n* = 5 *p* = 0.0670) (Fig. 3U). In Fig. 3U, we selected the P18 stage because it represents the peak of photoreceptor degeneration and is also chosen for organotypic retinal cultures. In *wt* retinas at P18, the density of cone photoreceptors was higher in both dorsal and ventral parts compared to *rd2* P18 retinas (Fig. 3V). When comparing different retinal regions in *wt* mice, we observed that at P18, the density of cone photoreceptors was highest in the middle (ON: 1.33 ± 0.04 SEM *n* = 3 *p* = 0.1721, middle: 1.43 ± 0.01 SEM *n* = 3 *p* = 0.5016, periphery: 1.28 ± 0.04 SEM *n* = 3 *p* = 0.0387) (Fig. 3U). (*For the technique used to calculate the percentage and density of cone photoreceptors, please see Additional files* 1, 2, and 3* in the supplementary information)*.

To detect dying cone photoreceptors, we performed a TUNEL assay and then colocalized it with a cone marker. The percentage of total TUNEL-positive cells increased dramatically in *rd2* retinas at P18 day (*wt*: 0.15 ± 0.02 SEM *n* = 4, *rd2*: 1.51 ± 0.10 SEM *n* = 4 *p* < 0.0001). Cone-Arrestin (CAR) staining colocalized with TUNEL, showed that cone cell death was significantly increased in *rd2* P18 retinas both in percentage (*wt*: 0.04 ± 0.00 SEM *n* = 4, *rd2*: 0.92 ± 0.10 SEM *n* = 4 *p* = 0.0002) and density per 10 µm (*wt*: 0.01 ± 0.00 SEM *n* = 4, *rd2*: 0.24 ± 0.03 SEM *n* = 4 *p* = 0.0004) (Fig. 3W–Y). *(For regional examination of cone photoreceptor degeneration in rd2 P18 retinas, please see Additional file* 4* in the supplementary information).*

To understand the extent of cone photoreceptor death, we compared it to the total number of photoreceptor cell deaths. Accordingly, the percentage of dead cone photoreceptors in the *rd2* retina was higher than that of rod photoreceptors at the peak of photoreceptor (P18) (Fig. 3Z), accounting for 60.9% of total photoreceptor death.

### PARP inhibition preserves cone photoreceptors in *rd2* retinas

**Fig. 4:**
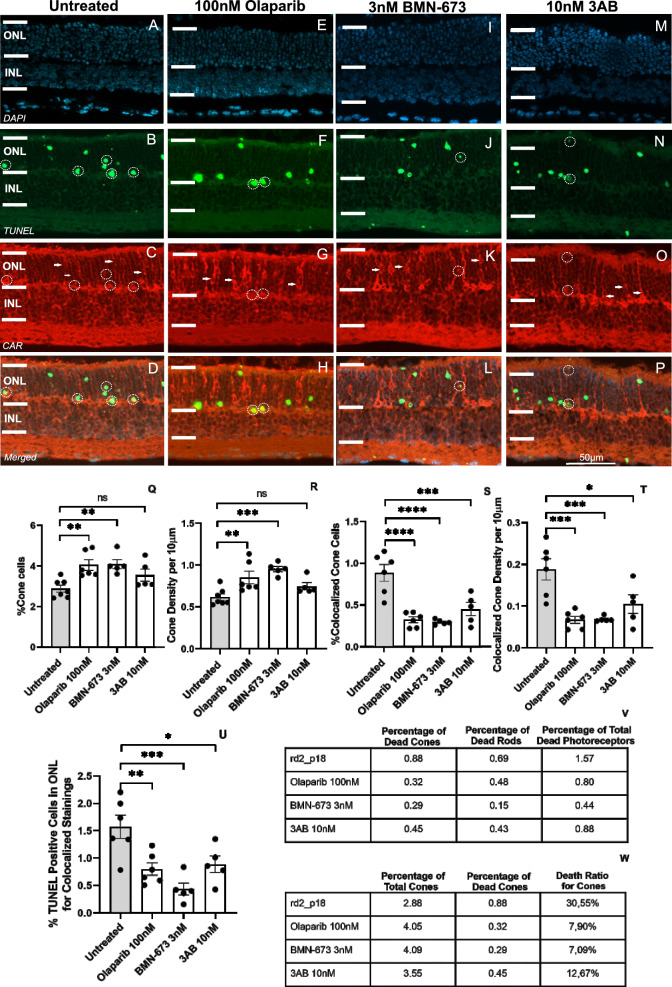
PARP inhibition improves cone photoreceptor survival in *rd2* organotypic retinal cultures. The TUNEL-positive cells (green) in *rd2* retinal organotypic cultures treated with and without PARP inhibitors (**B, F, J, N**), DAPI (blue) was used as a nuclear counterstain (**A, E, I, M**); CAR staining for cones (red) (**C, G, K, O**) and merged images (**D, H, L, P**). Circles indicate colocalized cones stained with TUNEL, CAR, and in the merged image. CAR staining indicated a significantly increased percentage and density of cone photoreceptors in the ONL for 100 nM Olaparib and 3 nM BMN-673 treated groups (**Q, R**). Both the percentage and density of TUNEL-CAR colocalization indicated a significant reduction of photoreceptor death in all treatment groups (**S, T**). The TUNEL-positive cells significantly reduced in *rd2* retinal organotypic cultures treated with PARP inhibitors (**U**). Comparison of rod, cone and total photoreceptor cell death in *rd2* retinal organotypic cultures treated with PARP inhibitors (**V**). The percentage of cone cell death in total cones in *rd2* retinal organotypic cultures treated with PARP inhibitors (**W**). Arrows indicate representative cone photoreceptors, and circles indicate representative dead cone photoreceptors. The images shown are representative for observations on at least three different specimens for each genotype/treatment condition. *n* ≥ 5, significance levels: ns > 0.05, *p < 0.05, **p < 0.01, ***p < 0.001, ****p < 0.0001, ANOVA test, and Dunnett’s test for multiple comparisons

To investigate the potential of PARP inhibitors in preserving cone photoreceptor viability, we assessed both the percentage and density of cone photoreceptors in *rd2* organotypic retinal cultures treated with the most effective concentrations of Olaparib (100 nM), BMN-673 (3 nM), and 3AB (10 nM). In determining these most effective doses, we selected the doses that best reduced PAR accumulation and percent photoreceptor death for all three PARP inhibitors. As a result, we found a significant increase in both the percentage of cone photoreceptors (untreated: 2.88 ± 1.16 SEM *n* = 7, 100 nM Olaparib: 4.05 ± 0.25 SEM *n* = 6 *p* = 0.0004, 3 nM BMN-673: 4.09 ± 0.22 SEM *n* = 5 *p* = 0.0004, 10 nM 3AB: 3.55 ± 0.29 SEM *n* = 5 *p* = 0.1407) and cone photoreceptor density (untreated: 0.61 ± 0.03 SEM *n* = 6, 100 nM Olaparib: 0.85 ± 0.07 SEM *n* = 5 *p* = 0.0070, 3 nM BMN-673: 0.95 ± 0.03 SEM *n* = 5 *p* = 0.0005, 10 nM 3AB: 0.74 ± 0.04 SEM *n* = 5 *p* = 0.2077) (Fig. 4A–P, Q, R) in the Olaparib and BMN-673 groups*.* Based on the results of Cone-Arrestin (CAR) staining performed in colocalization with TUNEL staining, a significant reduction in cone photoreceptor cell death was observed in all treatment groups. Both percentage of colocalized cone photoreceptors (untreated: 0.88 ± 0.09 SEM n = 6, 100 nM Olaparib: 0.32 ± 0.03 SEM *n* = 6 *p* < 0.0001, 3 nM BMN-673: 0.29 ± 0.01 SEM *n* = 5 p < 0.0001, 10 nM 3AB: 0.45 ± 0.07 SEM *n* = 5 *p* = 0.0009) and density per 10 µm (untreated: 0.19 ± 0.02 SEM *n* = 6, 100 nM Olaparib: 0.07 ± 0.00 SEM *n* = 6 *p* = 0.0002 = , 3 nM BMN-673: 0.07 ± 0.00 SEM *n* = 5 *p* = 0.0005, 10 nM 3AB: 0.10 ± 0.02 SEM *n* = 5 *p* = 0.0102) were reduced (Fig. 4S,T), indicating surviving cone photoreceptors.

Next, we aimed to assess the ratio of decreased cone and rod photoreceptor degeneration within the treatment groups. To achieve this, we evaluated three key parameters: the percentage of TUNEL and cone photoreceptor colocalization, the density of TUNEL and cone photoreceptor colocalization, and the percentage of total TUNEL-positive cells in the photoreceptor layer. Our results showed that PARP inhibition significantly reduced cone photoreceptor cell death in the treated groups (Fig. 4S–W). 100 nM Olaparib, 3 nM BMN-673 and 10 nM 3AB treatments preserved cone photoreceptors (untreated: 0.88 ± 0.09 SEM, *n* = 6; 100 nM Olaparib: 0.32 ± 0.03 SEM, *n* = 6, *p* = 0.0051; 3 nM BMN-673: 0.29 ± 0.01 SEM, *n* = 5, *p* = 0.0002; 10 nM 3AB: 0.45 ± 0.08 SEM, *n* = 5, *p* = 0.0170) (Fig. 4S). We observed a significant decrease in photoreceptor cell death in all treatment groups (untreated: 1.57 ± 0.20 SEM, *n* = 6; 100 nM Olaparib: 0.80 ± 0.11 SEM, *n* = 6, *p* = 0.0051; 3 nM BMN-673: 0.44 ± 0.11 SEM, *n* = 5, *p* = 0.0002; 10 nM 3AB: 0.88 ± 0.15 SEM, *n* = 5, *p* = 0.0170). To assess the rod photoreceptor death, we used the total number of photoreceptors according to the formulation as described in our previous study [35] and TUNEL-positive cone photoreceptors in ONL of each retina at the peak of degeneration. Intriguingly, we observed that the percentage of cone death (0.88%) numerically exceeded that of rod death (0.69%) (Fig. 4V). Subsequently, we assessed the efficacy of PARP inhibitors in reducing cone death. To do this, we compared the percentage of TUNEL-cone photoreceptor colocalization with the percentage of total cone photoreceptors in the same area. Our results showed that the BMN-673 group exhibited the lowest cone photoreceptor cell death (Fig. 4W).

### PARP inhibition decreases Müller cell activation in *rd2* organotypic retinal cultures

**Fig. 5:**
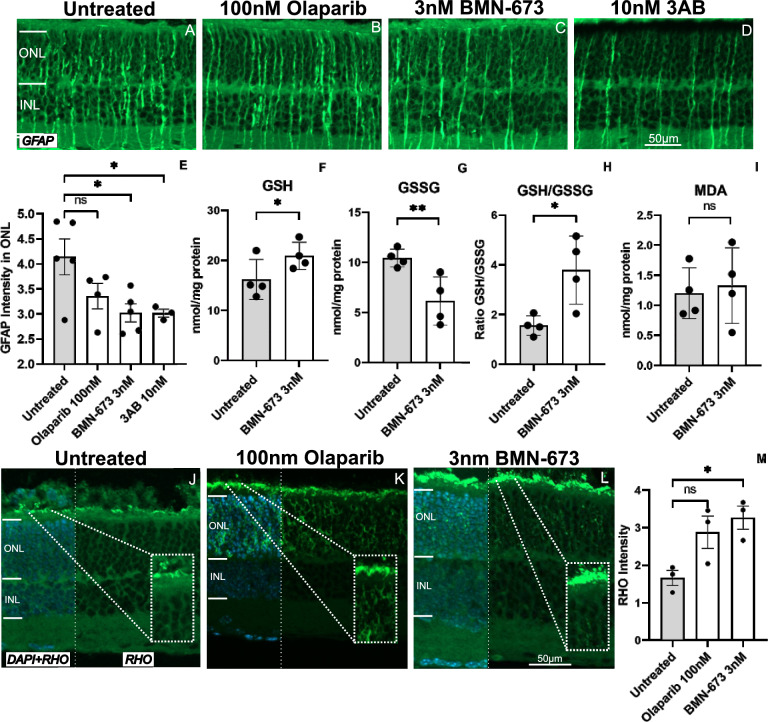
PARP inhibition reduces GFAP level in *rd2* retinas (**A–E**). Immunostaining for GFAP in *rd2* retinas and treated with 100 nM Olaparib, 3 nM BMN-673, and 10 nM 3AB (**A–D**). GFAP expression was significantly decreased for 3 nM BMN-673 and 10 nM 3AB treated groups (**E**). PARP inhibition decreases oxidative stress (**F–I**). According to the GSH and GSSG levels, GSH levels were increased significantly, and GSSG levels were decreased significantly in the BMN-673-treated group compared to the untreated *rd2* group (**F, G**). The GSH/GSSG ratio was increased significantly in retinas treated with BMN-673 (**H**). MDA retinal concentration did not change significantly after treatment with the PARP inhibitor BMN-673 (**I**). PARP inhibition improved the development of outer segment formation (**J–M**). Represented images of immunostaining images for Rhodopsin expression in green and DAPI in blue in *rd2* retinas and treated *rd2* retinas with 100 nM Olaparib and 3 nM BMN-673. Rhodopsin expression was increased in the 3 nM BMN-673 treated group (**M**). The images shown are representative for observations on at least three different specimens for each genotype. *n* ≥ 3, significance level: ns > 0.05, *p < 0.05, **p < 0.01, Unpaired *t*-test, ANOVA test, and Dunnett’s test for multiple comparisons

Retinal degeneration triggers the activation of Müller glial cells, which is associated with the expression of glial fibrillary acidic protein (GFAP) [40]. Therefore, we also performed GFAP analysis to investigate Müller cell activation in *rd2* retinas. Accordingly, GFAP expression was observed in *rd2* retinas treated with 100 nM Olaparib, 3 nM BMN-673, and 10 nM 3AB and untreated *rd2* retinas. GFAP expression was downregulated only in *rd2* retinas treated with 3 nM BMN-673 and 10 nM 3AB (untreated: 4.14 ± 0.35 SEM *n* = 5, 100 nM Olaparib: 3.35 ± 0.25 SEM *n* = 4 *p* = 0.1352, 3 nM BMN-673: 3.02 ± 0.17 SEM *n* = 5 *p* = 0.0192, 10 nM 3AB: 3.01 ± 0.07 SEM *n* = 3 *p* = 0.0410) (Fig. 5A–E).

### PARP inhibition decreased oxidative stress markers in *rd2* retinas

Glutathione (GSH) is a potent intracellular antioxidant that protects the cell from oxidative stress, which may be increased by factors such as various mutations or adverse environmental conditions. It can be found in the cell in both reduced (GSH) and oxidized (GSSG) forms [41]. Previous studies have shown that GSH levels decrease in various diseases, such as Leber’s hereditary optic neuropathy, diabetic retinopathy (DR), AMD, and glaucoma, in which retinal degeneration is observed [42]. We continued with only Olaparib and BMN-673 for further mechanistic studies (oxidative stress and CD9 expression). Both inhibitors are repurposed drugs and had already demonstrated greater effectiveness than 3AB in our previous experiments. In this study, we measured GSH and GSSG levels in untreated *rd2* mouse retinas of day P18 and *rd2* mouse retinas treated with BMN-673. We also determined the ratio of GSH to GSSG, one of the frequently used markers of cellular oxidative stress. Accordingly, GSH level was increased significantly in the BMN-673 treatment group (*rd2*: 16.19 ± 2.41 SEM *n* = 4, BMN-673: 20.88 ± 2.41 SEM *n* = 4 *p* = 0.05) (Fig. 5F). On the other hand, the oxidized glutathione (GSSG) levels were significantly decreased in BMN-673 treated retinas compared to untreated *rd2* retinas (*rd2*: 10.44 ± 1.28 SEM *n* = 4, BMN-673: 6.15 ± 1.28 SEM *n* = 4 *p* = 0.0078) (Fig. 5G). In addition, the GSH/GSSG ratio was increased significantly in the treatment group (*rd2*: 1.56 ± 0.71 SEM *n* = 4, BMN-673: 3.78 ± 0.71 SEM *n* = 4 *p* = 0.0101) (Fig. 5H).

Malondialdehyde (MDA) is one of the end products of lipid peroxidation. It is known to increase in correlation with oxidative stress [43] and is also toxic in itself. In this study, we also measured MDA levels in untreated *rd2* mouse retinas of day P18 and *rd2* mouse retinas treated with BMN-673. There was no statistically significant difference between untreated *rd2* and treated with BMN-673 group (*rd2*: 1.20 ± 0.37 SEM *n* = 4, BMN-673: 1.32 ± 0.37 SEM *n* = 4 *p* = 0.3753) (Fig. 5I).

### PARP inhibition improved rod outer segment development

As previously demonstrated, *rd2* mice exhibit impaired outer segment development leading to progressive photoreceptor degeneration [3, 44]. In a separate study, healthy and *rd2* retinas were compared to assess outer segment formation and a significant reduction in the presence of rhodopsin was found in *rd2* [45]. In this study, we investigated the development of outer segment formation using ex vivo cultures derived from the *rd2* mice. Specifically, we examined the effect of 100 nM Olaparib and 3 nM BMN-673 treatments on outer segment development in *rd2* retinas, focusing on rhodopsin expression. Our findings revealed a statistically significant increase in the density of rhodopsin staining in *rd2* organotypic retinal cultures following treatment with 3 nM BMN-673 (untreated: 1.66 ± 0.19 SEM *n* = 3, 100 nM Olaparib: 2.88 ± 0.43 SEM *n* = 3 *p* = 0.0660, 3 nM BMN-673: 3.27 ± 0.30 SEM *n* = 3 *p* = 0.0231) (Fig. 5J–M).

### PARP inhibition decreases augmented extracellular vesicles in *rd2* retinas

**Fig. 6:**
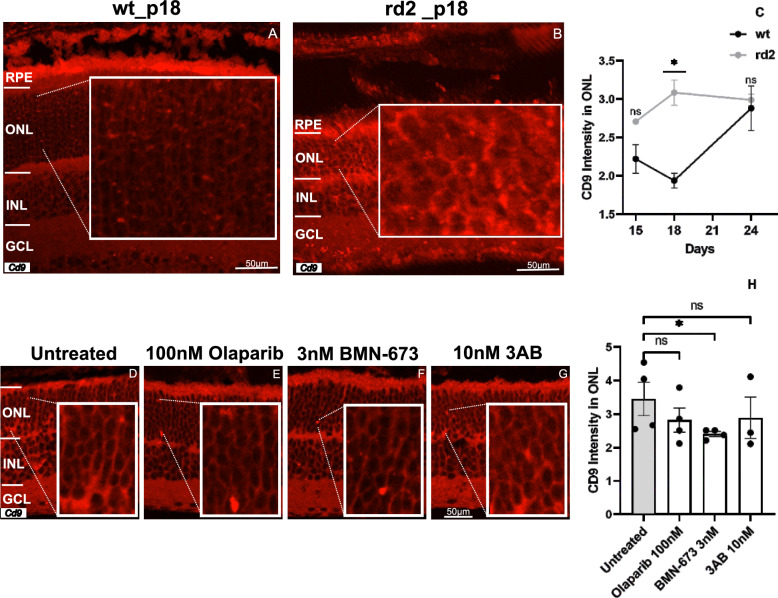
CD9 expression is increased in *rd2* retinas. Representative images of CD9 expression in different layers of *wt* retinas (**A**) and *rd2* (**B**) at P18. CD9 expression was increased statistically in *rd2* retinas in the ONL at P18 (**C**). PARP inhibition changed CD9 level in in vitro organotypic retinal cultures derived from *rd2* retina. Representative images of CD9 staining in untreated *rd2* and treated *rd2* with Olaparib, BMN-673, and 3AB PARP inhibitors (**A, B, D, E, F, G**), BMN-673 treated group showed significant difference. However there was a decrease in all three treatment groups compared to the control group (**D–H**). The images shown are representative for observations on at least three different specimens for each genotype/treatment condition. *n* ≥ 3, significance levels: ns > 0.05, *P < 0.05. ANOVA test and Kruskal–Wallis test for multi-comparison

Previously, we demonstrated the involvement of extracellular vesicles (EVs) in the process of inherited photoreceptor degeneration in a *Pde6* mutation by CD9 expression [46]. CD9 is a protein belonging to the tetraspanin family and is observed on the surface of cell membranes and extracellular vesicles [47, 48]. CD9 expression was observed in the ONL and GCL of *rd10* retinas [41]. To investigate the potential involvement of EVs, we examined the expression of CD9 in *rd2* and corresponding *wt* retinas at P15, P18, and P24. Our results showed a significant difference in CD9 expression within ONL of *rd2* retinas at P18 (*wt*: 1.93 ± 0.09 SEM *n* = 3, *rd2*: 3.08 ± 0.16 SEM *n* = 5 *p* = 0.0357) (Fig. 6A–C). For the expression of CD9 in the GCL, please see *Additional file *5* in the supplementary information.*

In our previous publication, we reported that CD9 is expressed in the ONL and GCL of *rd10* retinas, a model of rod degeneration, and that CD9 expression is reduced in the ONL and GCL layers of Olaparib-treated *rd10* retinas compared to untreated *rd10* retinas [46]. Here, we hypothesized that PARP inhibition may change behaviour of EVs in *rd2* retinas. Therefore, we analyzed CD9 expression in *rd2* organotypic retinal cultures treated with three PARP inhibitors. The result showed that a decrease in CD9 expression in *rd2* retinas treated with 3 nM BMN-673 compared to untreated *rd2* retinas (untreated: 3.45 ± 0.49 SEM *n* = 4, 100 nM Olaparib: 2.82 ± 0.35 SEM *n* = 4 *p* = 0.2059, 3 nM BMN-673: 2.40 ± 0.07 SEM *n* = 4 *p* = 0.0481, 10 nM 3AB: 2.89 ± 0.61 SEM *n* = 3 *p* = 0.1367) (Fig. 6D–H).

## Discussion

In this study, we demonstrated the neuroprotective effects of Olaparib, BMN-673, and 3AB on cone and rod photoreceptor degeneration. Although the absence of photoreceptor OSs due to *Prph2* mutation leads to the loss of both rods and cones [7, 49–51], their sensitivity to stress conditions was unknown. Our findings highlighted a cone-dominant photoreceptor degeneration in *rd2* retinas. In addition, we showed that cone photoreceptors were protected by BMN-673, Olaparib, and 3AB, which have previously been shown to protect rod photoreceptors. Furthermore, PARP inhibition positively contributed to OS formation in *rd2* retinas. Among PARP inhibitors tested in this study, BMN-673 was the most effective in preventing cone and rod photoreceptor cell death in *rd2* retinas. We found novel mechanisms and consequences of PARP-dependent neuroprotection of rod and cone degeneration, such as reduction of CD9 expression and Müller cell activity, as well as enhancement of antioxidant GSH marker and reduction of GSSG oxidative stress marker.

*PRPH2* gene mutations are a common cause of retinal dystrophies, together with AMD [52] and diabetic retinopathy (DR) [53]. Recent clinical studies have shown that cone function is more impaired than rod function in patients who have *PRPH2* mutations [54–56]. A recent study has shown that rods and cones are affected differently in various *Prph2* mutations depending on the mutations. Mutations in the second intradiscal loop, where protein folding and oligomerization are controlled, cones have been less affected comparing to rods [57]. A previous study showed that cone photoreceptor density in the retina of *wt* mice was higher in the middle part of the retina [58]. Similarly, in this study, we divided the retina into three regions: optic nerve, middle, and periphery. In wild-type retinas, we found that the cone photoreceptor density was the highest in the middle regions, consistent with the findings of Volland et al., 2015. We showed that cone photoreceptor density was significantly reduced in *rd2* retinas compared to the corresponding *wt.*

Previously, we have shown PARP-dependent photoreceptor cell death in different mouse and rat models [19, 59]. Consequently, PARP inhibition protected rod photoreceptors in different mouse models of inherited retinal degeneration [36, 37, 45, 46, 59]. Although the neuroprotective effect of PARP is known, the mechanism of action is not yet well understood. One function of PARP enzymes is to maintain cell viability and genetic stability by repairing DNA strand breaks caused by mild and moderate genotoxic factors [60]. PARP-dependent cell death, PARthanatos, can result in toxicity due to PAR accumulation [61] or change in cellular energy metabolism due to over-consumption of NAD+ in the PARylation process [24, 25]. In addition to our previous studies in inherited retinal dystrophies [36, 45], other studies have implicated PARP activation in other neurodegenerative diseases [62], including retinal diseases such as AMD and DR [20, 63]. In our previous study, the well-known PARP inhibitor PJ34 exhibited a decrease in PAR levels, resulting in a reduction in photoreceptor cell death in *rd2* retinas [45]. In parallel, rhodopsin expression was improved when *rd2* retinas were treated with PARP inhibitors [45]. However, PJ34 is not a potent PARP inhibitor. Since potent PARP inhibitors have been developed, we chose two approved inhibitors to facilitate translation into clinical trials for peripherin-2 related retinal diseases. In this study, the inhibition of PARP with 100 nM Olaparib and 3 nM BMN-673 effectively reduced both cone and rod photoreceptor cell death in *rd2* retinas. Furthermore, our results demonstrated that three PARP inhibitors, Olaparib, BMN-673, and 3AB, decreased PARylation and photoreceptor cell death in *rd2* retinas. It is crucial to note that within the nanomolar range, 3-aminobenzamide may inhibit mono ADP-ribosylation [39], suggesting a potentially more effective role in the inhibition of PARP activity in retinal degeneration. Nevertheless, additional studies are necessary to comprehend the interactions between different PARP inhibitors and the PARP enzyme.

Since cone photoreceptors are more susceptible to *rds* mutation-dependent stress conditions [64], the preservation of cones was profound compared to rods. Similarly, in a Stargardt model, an inherited retinal disease, it was shown that cone photoreceptors are much more sensitive to stress than rods [65]. Some clinical studies confirm that cone-rich foveal area is especially vulnerable to stress in AMD or Stargardt disease type 1 [66, 67]. In this study, the level of cone photoreceptor degeneration was numerically higher than rod photoreceptor degeneration in *rd2* retinas at the peak of degeneration. Interestingly, we observed that different PARP inhibitors had different effects on cone and rod degeneration. Indeed, we did not observe any protective effect of Olaparib on secondary cone degeneration in *rd1* model, a primary rod degeneration model [36]. In our previous study, 100 nM 3AB was the most effective PARP inhibitor in reducing PARylation and photoreceptor cell death [37]. However, in this study, BMN-673 proved more potent, particularly in reducing PARylation. This indicates that increased PARP activity in cone degeneration due to peripherin-2 mutations advocates for the potential use of PARP inhibition as a viable treatment for retinal stress conditions.

EVs are heterogeneous membrane-enclosed vesicles released from different cell types under physiological and pathological conditions [68, 69]. Previous studies have shown that EVs are associated with PARP activation in different diseases [46, 70, 71]. CD9 expressions are changed in different layers of the retina in the rod photoreceptor degeneration model, *rd10* mice [46]. CD9 is a member of the tetraspanin family and is highly enriched in EV membranes. CD9 expression increased in the choroid and photoreceptors. However, it decreased in the retinal pigment epithelium in *rd10* mice [46]. Here, we showed a significant increase in CD9 expression in the photoreceptor layer in *rd2* retinas compared to corresponding *wt* retinas at the peak of photoreceptor degeneration, and after the peak of degeneration, e.g., P15, P18, and P24. Furthermore, CD9 level was increased in the ganglion cell layer when we compared different layers at different time points in *rd2* retinas. Likewise, a notable reduction in the expression of CD9 expression was observed in the outer nuclear layer (ONL) when PARylation was inhibited in *rd*2 retinas.

Müller cells play several fundamental roles in supporting retinal homeostasis and modulating synaptic activity in the inner retina through the uptake and exchange of neurotransmitters [72]. In response to neuronal degeneration, Müller cells undergo morphological and functional changes known as reactive gliosis [73]. Müller glia cells play a pivotal role in the process of reactive gliosis, a stress response associated with retinal degenerative conditions, including AMD [74]. It is known that metabolic imbalance induces Müller cell reprogramming [75] and that prolonged exposure to this situation increases oxidative stress load [76], causing DNA damage and inducing PARP1 activity [77]. PARP1 can regulate the nuclear distribution of splicing factors in response to oxidative stress and, consequently, DNA damage [78]. Our results indicated that PARP inhibition reduced GFAP expression, which was upregulated in parallel with GSH oxidative stress markers. This may be a mechanism of PARP-dependent neuroprotection of cone and rod photoreceptors in *rd2* retinas.

Oxidative stress has emerged as a prominent factor contributing to retinal tissue damage, such as glaucoma, AMD, diabetic retinopathy (DR), and IRD [41]. Many studies on oxidative stress have been carried out in animal models of hereditary retinal degeneration, particularly in *rd1* and *rd10* mice [42, 43, 52, 79]. Oxidative stress and inflammation occur in a time-dependent manner in *rd2* mouse retina [47]. Glutathione (GSH), a crucial player in maintaining cellular viability, neutralizes reactive oxygen species (ROS)-like metabolites responsible for oxidative stress. This tripeptide component is an intracellular antioxidant and catalyzes the conversion of H_2_O_2_ to H_2_O by glutathione peroxidase [53]. GSH levels have previously been observed to peak around postnatal day 21 (P21) in *rd2* mouse retinas, marking a critical period of oxidative stress [47]. We investigated PARP inhibition’s effect on GSH levels and found a notable increase, suggesting reduced oxidative stress. BMN-673 treatment lowered oxidized glutathione (GSSG) in *rd2* retinas, increasing the GSH/GSSG ratio, a key oxidative stress marker. PARP inhibitors may help restore mitochondrial function [33, 34].

## Conclusion

In conclusion, our study revealed a significant reduction in both the density and distribution of cone photoreceptors within *rd2* retinas. Notably, targeted inhibition of PARP, particularly with the potent and FDA-approved PARP inhibitor BMN-673, emerged as a promising strategy to preserve cone photoreceptors by reducing oxidative stress, EVs, and Müller cell activity in *rd2* retinas. These findings underscore the potential therapeutic significance of PARP inhibitors in the context of rod-parallel cone degeneration and secondary cone degeneration and suggest that they may be promising therapeutic targets for photoreceptor viability.

## Materials and methods

### Experimental animals

*Rd2* and *wt* animals at P9 (organotypic retinal culture) and P15, P18, and P24 (for in vivo preparations) were used irrespective of gender. Animals were housed under standard white cyclic lighting and had free access to food and water. All procedures were performed under the ARVO statement for the use of animals in ophthalmic and visual research and were approved by the state authorities (Regierungspraesidium, Tübingen) and conducted under the supervision of the University of Tübingen’s facility for animal welfare (Einrichtung für Tierschutz, Tierärztlichen Dienst und Labortierkunde) directed by Dr. Franz Iglauer, and by the CEU Cardenal Herrera Universities Committee for Animal Experiments (approval code 2020/VSC/PEA/0094).

### Organotypic retinal cultures

Organotypic retinal culture, a well-established method, was used for the treatment [36]. Briefly, the eyes were enucleated and incubated for 15 min at 37 °C in pre-warmed 0.12% proteinase K (ICN Biomedicals Inc., OH, USA; 193,504). Proteinase K was used to remove sclera from retinal pigment epithelium (RPE), and then 10% Fetal Calf Serum (FCS; PAN Biotech GmbH, Aidenbach, Germany; P30-3701) was used to inhibit Proteinase K activity. Eyes were washed with serum-free R16 basal medium (Thermo Scientific, Rockfort, Illinois, USA; 07490743A), and the cornea, sclera, lens, and choroid were removed aseptically and carefully under the microscope (Zeiss, Stemi 2000-C Stereo Microscope, Carl Zeiss Microscopy GmbH, Jena, Germany). Only the retina with RPE attached remained. The eye cup was cut into four wedges to spread flat like a clover-leaf and was transferred to a culture membrane insert (Millipore, Carrigtwohill, Cork, Ireland; PIHA03050) with the photoreceptor-side down and RPE-side up. The inserts were transferred into the six-well plates for the incubation and treatment. Explants were incubated in R16 medium with supplements at 37 °C in a humidified 5% CO_2_ incubator between P9 from P18, the peak of degeneration. For the first two days, no treatment was applied. After the first two days, treatments with different concentrations of Olaparib (Lynparza), BMN-673 (Talazoparib), and 3AB (3-aminobenzamide) were applied for seven days (P11-P18). Moreover, the culture medium was changed every two days. Olaparib, BMN-673, and 3AB were dissolved in dimethyl sulfoxide (DMSO; Sigma–Aldrich, Hamburg, Germany; D2650) and diluted in R16 medium with supplements. The same concentrations of DMSO by diluting with culture medium were added to the controls. The culture was finished at the peak of degeneration to analyze the neuroprotective effect of the treatments.

### Fixation and sectioning

Both explant cultures and eyes obtained from the in vivo study were fixed at room temperature for one hour in 4% paraformaldehyde (PFA) (Poysciences, Warrington PA, USA) in 0.1 M phosphate buffer (PB., pH 7.4) containing 0.2 M sucrose. The difference between in vivo and in vitro fixation is the hygiene procedure. Aseptic conditions must be used for in vitro organotypic retinal cultures. After fixation, tissues were washed for 10 min in phosphate buffer saline (PBS., pH 7.4) For cryoprotection, they were incubated in 10% sucrose solution for 10 min, 20% sucrose solution for 20 min, and 30% sucrose solution in PB for at least 30 min. The retinas were frozen in boxes filled with Tissue-Tek O.C.T. Compound (Sakura Finetek Europe, Alphen aan den Rijn, Netherlands; 4583). 12 μm vertical tissue sections were prepared on a Leica CM3050S CryoMicrotome (Leica Biosystems, Wetzlar, Germany), thaw-mounted onto Superfrost Plus Object slides (R. Langenbrinck, Emmendingen, Germany; 03–0060). The slides were dried at 37 °C for one hour and stored at − 20 °C until use.

### TUNEL assay

The terminal deoxynucleotidyl transferase dUTP nick end labeling (TUNEL) assay was performed on cryosections to assess cell death. For this reason, an in situ cell death detection kit with fluorescein isothiocyanate as the reporter fluorochrome (Roche Diagnostics, Mannheim, Germany; Ref. No: 11684795910) was used. After labeling, the sections were mounted in Vectashield with 4′,6-diamidino-2-phenylindole (DAPI) as a nuclear counterstain (Vector Laboratories, Burlingame, California, USA; H-1200).

### PAR immunohistochemistry

PAR immunochemistry (3,3′-diaminobenzidine (DAB) staining) was performed with in vitro organotypic *rd2* organotypic retinal cultures at P18. Frozen sections were air-dried for one hour at 37 °C and washed in PBS for 10 min. To avoid non-specific background, quenching solution was applied, which included 30% H_2_O_2_, MeOH, and 0,1% phosphate-buffered saline with Tween-20 (PBST., pH 7.4). The sections were incubated with 10% normal goat serum (NGS) in PBS containing 0.1% Triton X-100 for one hour followed by an anti-PAR antibody (1:200; Enzo Life Sciences, Lörrach, Germany; ALX-804–220-R100) incubation for overnight at + 4 °C. After the sections were washed in PBS for 30 min, biotinylated secondary antibody (1:100, Vector Laboratories Inc., Burlingame, CA, USA; BA-9200; in 5% NGS in PBST) was diluted in 5% normal goat serum in 0.1% PBST, and the sections were incubated for 1 h at room temperature (R.T.) again, PBS washing was applied for 30 min and the slides were incubated in Vectastain Elite ABC kit (Vector Laboratories Inc., Burlingame, California, USA; PK-4000) for one hour at R.T. To produce the color reaction, DAB staining solution (0.05 mg/ml NH4Cl, 200 mg/ml glucose, 0.8 mg/ml nickel ammonium sulphate, 1 mg/ml DAB, 0.1 vol. % glucose oxidase in PB was applied equally, sections were incubated for 60–90 s, immediately rinsed with PB to stop the reaction, and covered by Aquatex (Merck, Darmstadt, Germany; 1.08562.0050).

### Immunofluorescence staining

Frozen sections were air dried one hour at 37 °C, washed in PBS for 10 min, and blocked with blocking solution containing 10% normal goat serum, 1% bovine serum albumin (BSA), and 0.1% Triton X in PBS for one hour at R.T. After this preincubation, sections were incubated overnight in primary antibody in blocking solution at + 4 °C. Primary antibody sources and dilutions are listed in Table 1. Subsequently, sections were rinsed in PBS for 30 min and incubated with anti-rabbit and anti-mouse IgGs, coupled with Alexafluor-568 (Invitrogen; dilution 1:250–1:750, Waltham, MA, USA, A11011) and Alexafluor-488 conjugated secondary antibody (Invitrogen; dilution 1:250–1:750, Waltham, MA, USA, A21121). Sections were covered with Vectashield mounting medium with DAPI (Vector, Burlingame, CA, USA) to visualize cell nuclei.

**Table 1:** Primary antibodies used in this study

Antigen	Species	Dilution	Company	Article number
GFAP	Conjugated	1:100	Abcam	ab190288
CD9	Rabbit	1:50	Abcam	ab92726
ConeArr	Rabbit	1:100	Merck-Milipore	AB15282
Rho	Mouse	1:50	Merck-Milipore	MAB5316

### Oxidative stress markers

Retinal explants untreated or treated with BMN-673 3 nM (*n* = 4) were homogenized in prechilled 0.2 M potassium phosphate buffer, pH 7.0. This homogenate was used to assay reduced and oxidized glutathione (GSH and GSSG) and malondialdehyde (MDA) concentrations. Samples were kept frozen (− 80 °C) until biochemical assays were performed.

The content of GSH and other tiol derivatives in the eye homogenate was quantified by the method of Reed et al. [80]. The concentration of MDA was measured by liquid chromatography according to a modification of the method of Richard et al. [81] as previously described [82]. The protein content of the samples was determined using the Bradford method [83].

### Microscopy and cell counting

The cultures were analyzed using Zeiss Axio Imager Z.2 ApoTome2 microscope, AxioCam 506 MRm camera, and Zeiss AxioVision 4.7 software in Z-stack and mosaic mode at 20 × magnification. Three slices were taken per picture with a slice distance of 14 μm for Z-stack mode. At least four different animals were analyzed for each genotype. For in vitro* rd2* organotypic retinal cultures, the retinal sections were collected and analyzed from different central parts of the retina. Three randomized fields at 20 × magnification closest to the optic nerve were analyzed and evaluated for each animal. Similarly, measurements and evaluations were performed on sections taken from in vivo preparation samples, covering at least three areas: near the optic nerve, middle, and periphery. For quantitative analysis, positive cells in the entire ONL of three cross-sections per organotypic retinal culture were manually counted. The percentage of positive cells was determined by dividing the absolute number of positive cells by the total number of ONL cells. The total number of ONL cells was estimated by dividing the ONL area by the size of a photoreceptor nucleus (17.3 μm^2^), as measured by DAPI staining. Photoreceptor rows were evaluated by counting the individual nuclei aligned in one ONL column every 200 μm, and then averaging these counts. Cone density was calculated by counting positive labelled somata per 10 μm of ONL.

### Analysis of percentages and densities of cone photoreceptors

We measured both the percentages and densities of cone photoreceptors. We did this using the Z-stack mode of the Zen Blue Edition 3.1 program in the AxioCam microscope. Thus, by dividing the ONL of the retina into ten sections in each preparation, we counted the cone photoreceptors in each section and obtained the total number of cone photoreceptors. When we evaluated the total number of cone photoreceptors over the total area, we obtained the cone photoreceptor percentage. When we evaluated the cone photoreceptor density over the line length we drew on the midline of the microscopic image, we obtained the cone photoreceptor density *(*Additional file 1 and 2*).*

### Statistics

Statistical analysis was performed using GraphPad Prism 8 software (GraphPad Sofware, La Jolla, CA, USA). Student’s t-test was used for a single comparison, and One-way Anova test with Bonferroni correction, two-way Anova test with Tukey correction, and Kruskal–Wallis test were used for multiple comparisons. Mean ± standard error of the mean (SEM) was used for values. The significance levels were as follows: *p < 0.05, **p < 0.01, ***p < 0.001, ****p<0.0001 . Additionally, Corel DRAW 2020 was used for image processing.

## Supplementary Information

Supplementary material 1. Method to obtain cone percentage and cone density data.

Supplementary material 2. Images from different layers of rd2 P18 retinal section.

Supplementary material 3. Video for TUNEL colocalized cone photoreceptors in rd2 P18 retina.

Supplementary material 4. Regional examination of cone photoreceptor degeneration in the rd2 P18 retina.

Supplementary material 5. CD9 expression in GCL for wt and rd2 retinas on different degeneration days.

## Data Availability

Data is provided within the manuscript or supplementary information files. (Additional file 1, 2, 3, 4, and 5).

## Abbreviations

*3AB*: 3-Aminobenzamide
*AIF*: Apoptosis-inducing factor
*AMD*: Age-related macular degeneration
*DR*: Diabetic retinopathy
*ERG*: Electroretinogram
*EV*: Extracellular vesicle
*GCL*: Ganglion cell layer
*GFAP*: Glial fibrillary acidic protein
*GSH*: Glutathione
*GSSG*: Oxidized glutathione
*IRD*: Inherited retinal degenerations
*MDA*: Malondialdehyde
*ONL*: Outer nuclear layer
*PAR*: PolyADP ribose
*PARP*: PolyADP ribose polymerase
*Prph2*: Peripherin-2
*RP*: Retinitis pigmentosa
*RPE*: Retinal pigment epithelium
