# Supplementary material for: PARP inhibition preserves cone photoreceptors in rd2 retina
Source: Acta Neuropathol Commun. 2025 Apr 1;13:68. doi: 10.1186/s40478-025-01982-5 (PMC11963520; doi:10.1186/s40478-025-01982-5)
Supplement: Supplementary file 1 — Supplementary material 1. Method to obtain cone percentage and cone density data. [file 40478_2025_1982_MOESM1_ESM.pdf]

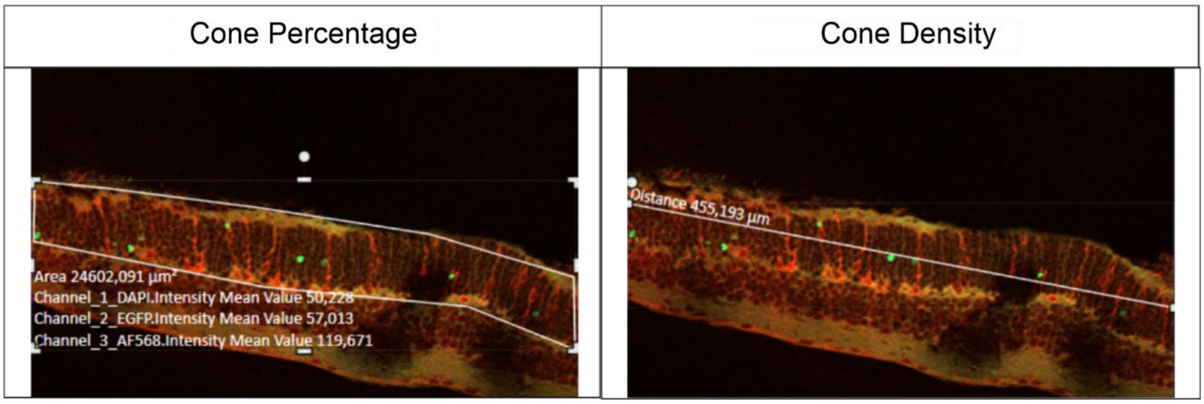

Additional file 1: Method to obtain cone percentage and cone density data. The percentage of cone photoreceptors in the retinal section was measured based on the total number of cones and the surface area of the section, while the cone photoreceptor density was calculated based on the total number of cones and the length of the line passing through the middle of the section.
