# Supplementary material for: PARP inhibition preserves cone photoreceptors in rd2 retina
Source: Acta Neuropathol Commun. 2025 Apr 1;13:68. doi: 10.1186/s40478-025-01982-5 (PMC11963520; doi:10.1186/s40478-025-01982-5)
Supplement: Supplementary file 2 — Supplementary material 2. Images from different layers of rd2 P18 retinal section. [file 40478_2025_1982_MOESM2_ESM.pdf]

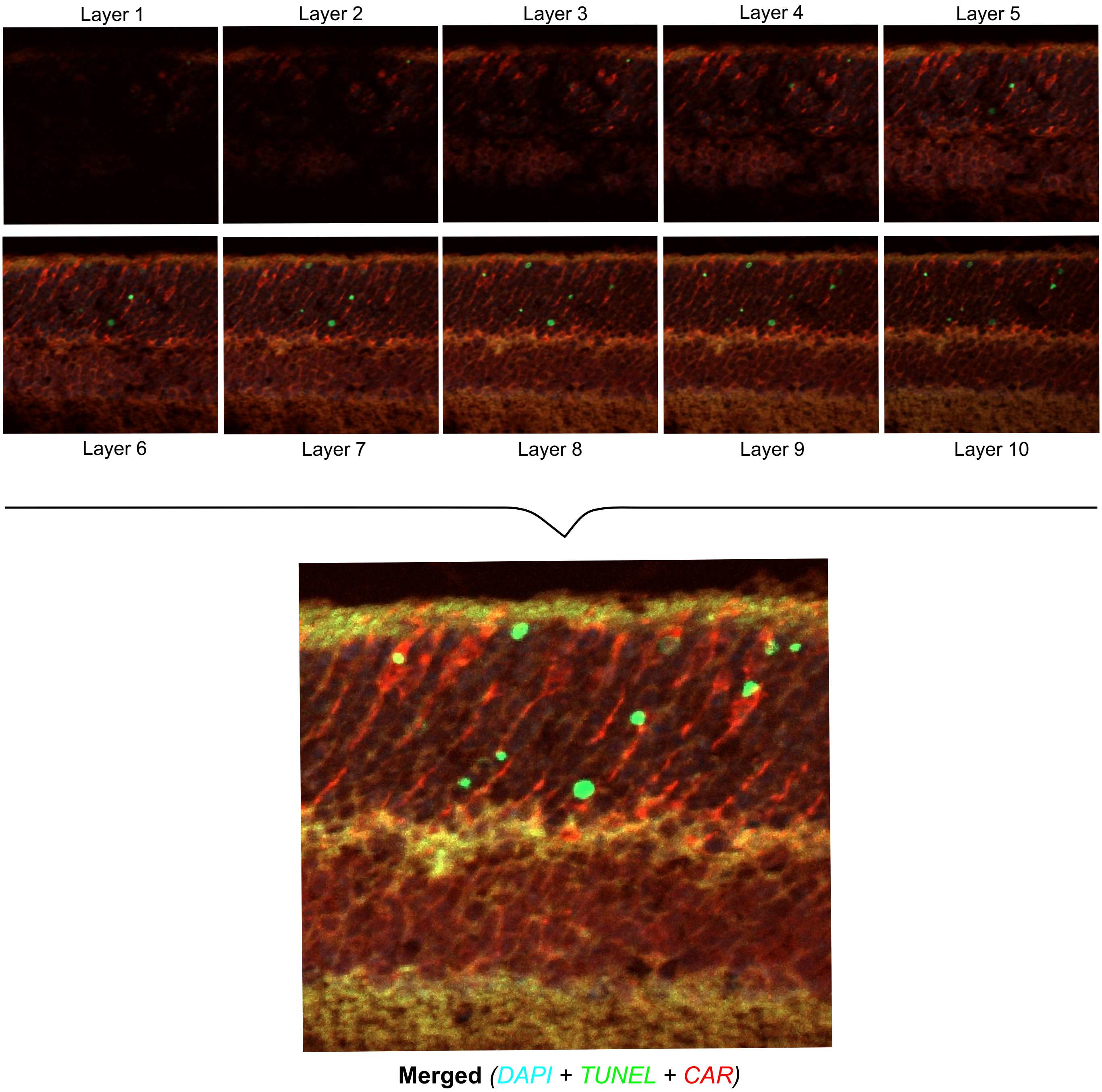

Additional file 2: Images from different layers of *rd2* P18 retinal section. Separate view of 10 layers of retinal section in Zen Blue 3.1 program Z-stack mode for detecting cone photoreceptors.
