# Supplementary material for: PARP inhibition preserves cone photoreceptors in rd2 retina
Source: Acta Neuropathol Commun. 2025 Apr 1;13:68. doi: 10.1186/s40478-025-01982-5 (PMC11963520; doi:10.1186/s40478-025-01982-5)
Supplement: Supplementary file 4 — Supplementary material 4. Regional examination of cone photoreceptor degeneration in the rd2 P18 retina. [file 40478_2025_1982_MOESM4_ESM.pdf]

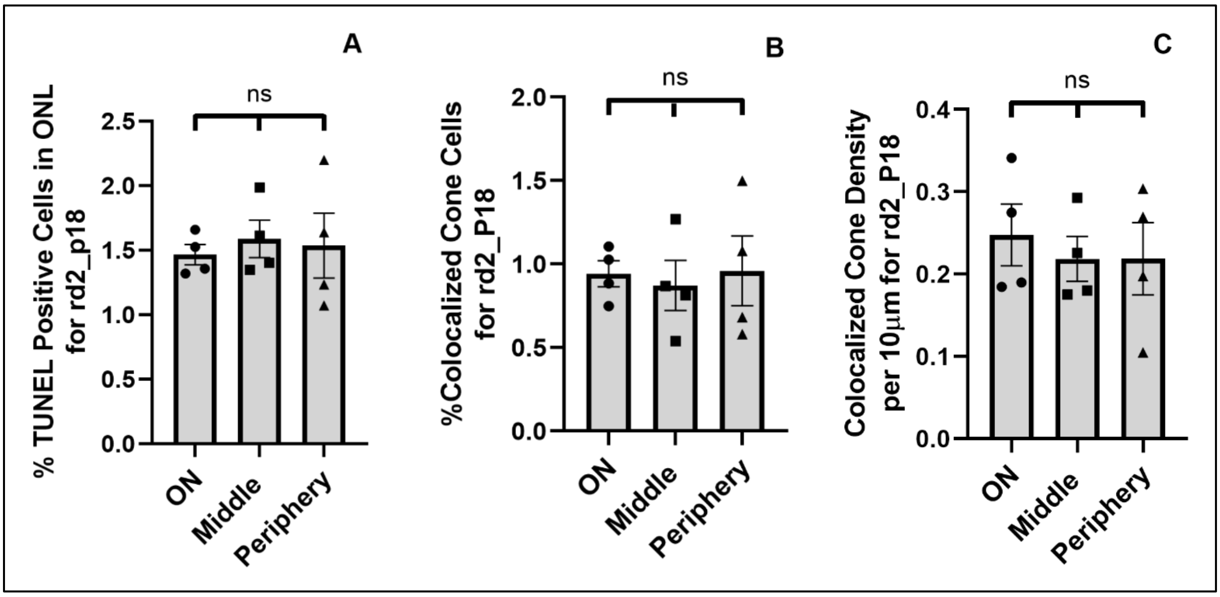

Additional file 4: Regional examination of cone photoreceptor degeneration in the *rd2* P18 retinas.

We also regionally examined TUNEL-positive cells for *rd2* P18. Although the result was not statistically significant, we found the maximum percentage of TUNEL positive cells in the middle region (ON: 1.47 ± 0.07 SEM n=4 p=0.8737, middle: 1.59 ± 0.14 SEM n=4 p=0.9558, periphery: 1.53 ± 0.25 SEM n=4 p=0.9756) On the other hand, in our analysis with CAR and TUNEL colocalized staining in *rd2* P18, although it was not statistically significant, the least percentage of colocalized cones (ON: 0.94 ± 0.08 SEM n=4 p=0.9471, middle: 0.87 ± 0.15 SEM n=4 p=0.9963, periphery: 0.96 ± 0.21 SEM n=4 p=0.9177) and colocalized cone density per 10μm (ON: 0.24 ± 0.04 SEM n=4 p=0.8457, middle: 0.22 ± 0.03 SEM n=4 p=0.8499, periphery: 0.22 ± 0.04 SEM n=4 p>0.9999) in the middle region *(Additional file 4A-C).*
