# Supplementary material for: PARP inhibition preserves cone photoreceptors in rd2 retina
Source: Acta Neuropathol Commun. 2025 Apr 1;13:68. doi: 10.1186/s40478-025-01982-5 (PMC11963520; doi:10.1186/s40478-025-01982-5)
Supplement: Supplementary file 5 — Supplementary material 5. CD9 expression in GCL for wt and rd2 retinas on different degeneration days. [file 40478_2025_1982_MOESM5_ESM.pdf]

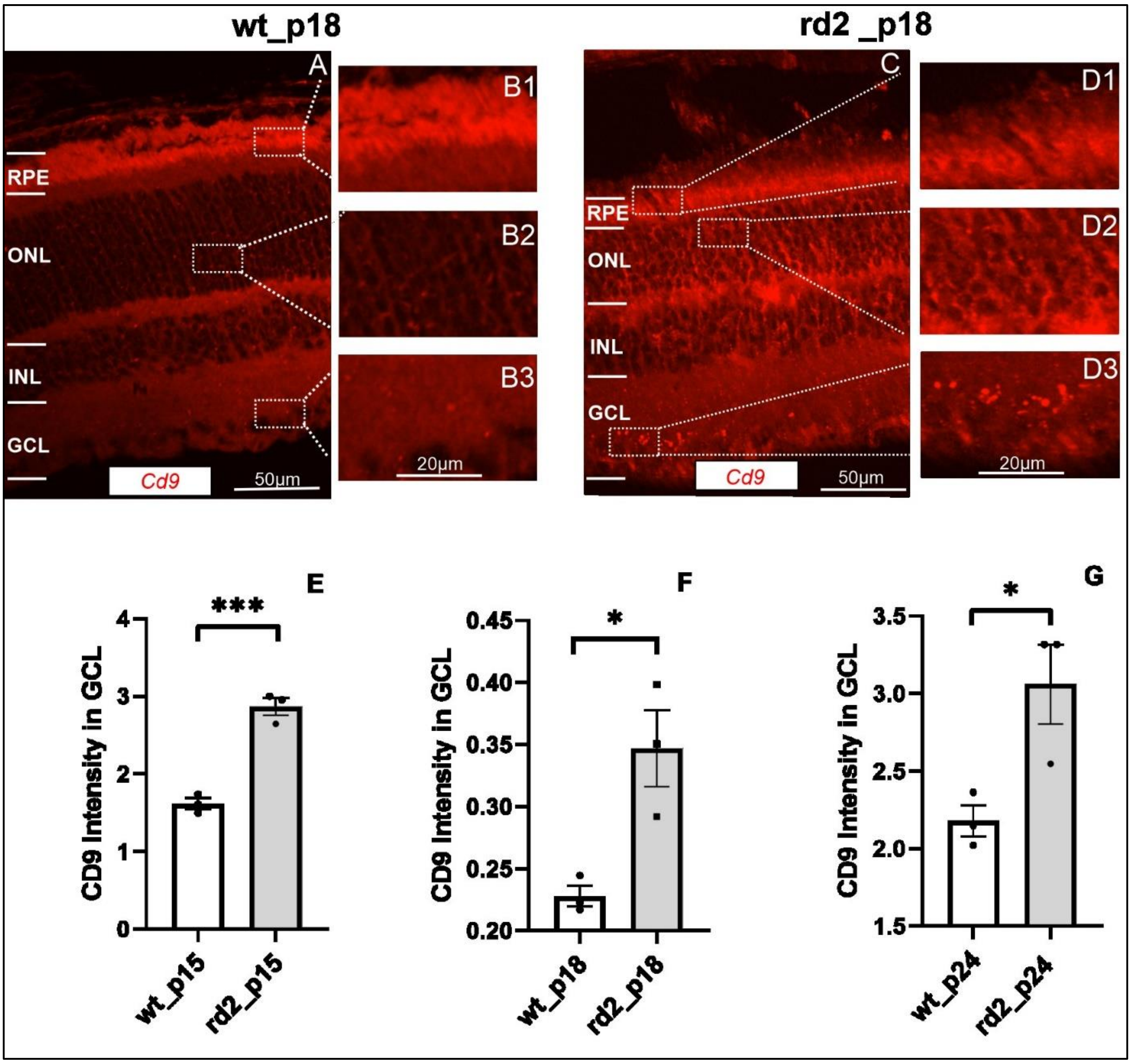

Additional file 5: CD9 expression in GCL for wt and rd2 retinas on different degeneration days.

An increase of CD9 expression was observed in the GCL of the rd2 retina at P15 (wt: 1.61 ± 0.07 SEM n=3, rd2: 2.86 ± 0.11 SEM n=3 p=0.0007) at P18 (wt: 0.22 ± 0.01 SEM n=3, rd2: 0.34 ± 0.05 SEM n=3 p=0.0203) and at P24 (wt: 2.17 ± 0.09 SEM n=3, rd2: 3.06 ± 0.25 SEM n=3 p=0.03) (Additional file 5B3,D3,E-G).

In order to better examine GCL at P18, we took our microscope images of the GCL at a magnification of 63X. We do not have 63X microscope pictures of days P15 and P24. Since our examinations for ONL are generally at 20X, we have 20X magnification microscope pictures for P15 and P24. At the same time we randomly selected measurement areas of different sizes (for ONL: 120*120, for GCL: 90*90) in the ImageJ program to make our statistical calculations. Therefore, there is no spaghetti diagram for GCL where we can examine the P15, 18 and 24 days together.
